# Differentiation of the body build and posture in the population of people with intellectual disabilities and Down Syndrome: a systematic review

**DOI:** 10.1186/s12889-024-17908-0

**Published:** 2024-02-08

**Authors:** Eliza Gaweł, Diana Celebańska, Anna Zwierzchowska

**Affiliations:** Institute of Sport Sciences, The Jerzy Kukuczka Academy of Physical Education in Katowice, Katowice, Poland

**Keywords:** Body composition, Flat foot, Spine, Overweight, Developmental disabilities, Cervical spine, Adolescent, Obesity

## Abstract

**Background:**

The aim of the study was to identify the variables of the internal compensatory mechanisms that differentiate the body build and posture of people with Down syndrome (DS) from the intellectual disability (ID) population. It was assumed that gaining knowledge in the abovementioned aspect will allow for a better understanding of the limitation of the kinesthetic abilities of people with ID and DS and simultaneously enable to optimize the process of planning and interventions to improve physical activity in this population with the adequate use of theirs strengths in the biomechanical and morphofunctional systems.

**Methods:**

The methodology of this systematic review was developed according to the PRISMA guidelines. A search of PubMed, EBSCO, Scopus databases was conducted to identify all studies on DS/ID and the body build and posture from 2003 to 2023.

**Results:**

395 articles were assessed to determine eligibility, while 22 studies met the inclusion criteria and were subjected to detailed analysis and assessment of their methodological quality. The differentiation of the body build and posture in DS population can be induced by both internal and external compensatory mechanisms. It is difficult to confirm the direct effect of the intrinsic variables that impact the body build and posture in the ID population, excluding people with DS.

**Conclusions:**

Compared to other ID, the intrinsic differences in the body build and posture in DS individuals were induced by gender, age, and level of ID. The tendency for diversity between DS and other ID populations in body build and posture may be determined by the presence of the third copy of chromosome 21 in DS group. Internal compensatory processes may be induced mainly by abnormalities in the structure of the cervical vertebrae and feet. IQ should not be used as the only variable that identifies the population of people with ID.

## Background

The harmonious and holistic morpho-functional development of the human body is strictly related to the ontogenetic evolution of the body posture that is, among others, determined by the anteroposterior spinal curvatures and the position of the pelvis [1]. Even though both of the aforementioned skeletal structures are believed to be the core variables of proper body posture [2], their position may be significantly influenced by the other segments of the human body, especially the lower limbs. As indicated in several studies [3, 4], among different musculoskeletal disturbances, biomechanical changes in the lower body’s structures i.e., knee joints and feet seem to significantly impact the development of further biomechanical disturbances in the upper segments of the body, including the pelvis and spinal cord, as part of body’s compensatory mechanisms [5, 6].

Compensatory mechanisms are known as intrinsic and independent processes that can be induced by both internal (musculoskeletal system) and external (physical activity) variables that can induce both internal and external compensatory processes in the biomechanical structures of the human body [7]. Available scientific literature indicates that the compensatory processess can occur both in healthy population and in people with disabilities [7]. However the compensatory disturbances are believed to be much more pronounced in people with disabilities compared to healthy individuals [8]. Furthermore, compensatory mechanisms are a cause-and-effect process that, in addition to the preliminary location, may induce further postural disturbances in the adjacent and/or opposite anatomical segments of the musculoskeletal system, thus intensifying musculoskeletal complaints and reducing the quality of life [6–8].

 Even though in the last few years researchers have become more focused on the issues of the body’s compensatory processes and their acute and long-term effects on everyday life [5], there is still a lack of studies addressing this issue in people with ID. This may be related to the incidence of diverse and concomitant developmental disorders that are frequently observed in this population. ID is believed to affect 1-3% of the global population, which is approximately 200,000,000 people worldwide (https://www.specialolympics.org). Moreover, this condition is characterized by neurodevelopmental disorders that include deficits in cognitive functions, adaptive function (conceptual, social, and practical domains) and disorders of the developmental period [9]. Consequently, people with ID exhibit more problems with sensory integration, poorer locomotor abilities, lower precision during everyday tasks [10], and disturbed coordination and balance skills [11]. Consequently, they frequently have more sedentary lifestyles and show a high incidence of obesity frequently [10, 12]. In addition, the abovementioned variables are related to the incidence of the disorders and deficits in the range of motion and body posture and may increase both musculoskeletal complaints and the biomechanical loads in the spinal curvatures, causing structural postural disturbances [13, 14].

Individuals with Down syndrome (DS) have become a specific part of the population of people with ID as they possess unique neurocognitive and neurobehavioral profiles that emerge in specific developmental periods and are distinct compared to other ID [15]. Moreover, individuals with DS are characterized by physical and motor proximodistal development that deviates from the typical cephalocaudal developmental model and is related to the process of the evolution of the locomotor system from the center of the body to its periphery [16], significantly affecting both the growth of the musculoskeletal system and body fat distribution. In addition, proximodistal development may influence motor skills and, as in people with ID, may cause several deficits in the biomechanical system that impact the quality of everyday life.

Individuals with ID and DS have already been the subject of previous qualitative analyses. To the best of the authors' knowledge, no study has simultaneously examined and compared those two populations in the aspects of the intrinsic compensatory mechanisms and developmental differences in body build and posture. Given the abovementioned findings and the gap in the available scientific literature, it seems justified to perform additional research to evaluate the abovementioned issues. Therefore, the aim of this systematic review (qualitative analysis) was to identify the variables of the internal compensatory mechanisms that differentiate the body build and posture of people with DS from the population of people with ID. It was assumed that gaining knowledge in the abovementioned aspect will allow for a better understanding of the limitation of the kinesthetic abilities of people with ID and DS and simultaneously enable to optimize the process of planning and interventions to improve physical activity in this population with the adequate use of theirs strengths in the biomechanical and morphofunctional systems.

## Methods

### Study design

The methodology of this systematic review was developed according to the Preferred Reporting Items for Systematic Reviews and Meta-Analyses (PRISMA) guidelines [17].

### Inclusion and exclusion criteria

In this systematic review, inclusion criteria for the study were: (a) cross-sectional study, case-control study, and cohort study; (b) males and females with DS or at least mild ID (ID individuals); (c) no mixed disabilities in the study group; (d) average age of study participants > 10 years, (e) no health condition except DS or at least mild ID (ID individuals).

The exclusion criteria were: (a) article type different than cross-sectional, case-control; and cohort study (b) physical, hearing or visual impairments; (c) study group including both DS and ID individuals; (d) poor methodological design; (e) average age <10 years or > 50 years; (f) DS or ID individuals not being the main aim of the study (validation of methods of assessment/indicators); (g) no full text available; (h) manuscript written in a language other than English.

### Literature search

A search of electronic databases (PubMed, EBSCO, Scopus) was conducted by two authors (EG, AZ) to identify all studies on DS/ID and the body build and posture from 2003 to 2023. The following methods were used: (a) data mining, and (b) data discovery and classification. As a prerequisite, all studies were performed in ID populations including males and females (mean age of the study group > 10 years). Search terms were combined by Boolean logic (AND/OR) in PubMed, EBSCO, and SCOPUS databases. The search was undertaken using the following 7 prioritized keyword combinations in English: ‘Down syndrome’, ‘intellectual disability’, ‘spinal curvatures’, ‘body posture’, ‘body composition’, ‘foot’, and ‘anthropometry’. Furthermore, two authors (EG, AZ) with expertise in the development of body build, posture, composition, and ID, including DS, reviewed the reference lists of the included studies and screened Google Scholar to find additional studies. The corresponding authors of the selected publications were also contacted directly if the crucial data were not available in the original articles.

### Methodological quality of the included studies (risk of bias)

The Joanna Briggs Institute (JBI) Critical Appraisal Checklist [18] for analytical cross-sectional study was used to evaluate the methodological quality of the included studies. The checklist is believed to be the newest and the most preferred tool for assessing the methodological quality (risk of bias) of analytical cross-sectional studies [18] and consists of 8 items (see Table 1) scored as ‘Yes’, ‘No’, ‘Unsure’, or ‘Not applicable’. A ‘Yes’ was assigned to the evaluated manuscript if the criterion was fulfilled, which simultaneously received a score of one. A ‘No’, ‘Unsure’, or ‘Not applicable’ was assigned to the evaluated manuscript if the criterion was not fulfilled, which simultaneously yielded a zero score. Each article was read and ranked by two independent investigators (EG, AZ). Moreover, an independent co-author (DC) was designated to resolve all discrepancies that could occur among investigators during the assessment. The methodological quality (risk of bias) was indicated by the total score (out of a possible 8 points), with the higher values representing better quality of the included publications.

**Table 1:** The assessment of the methodological quality of the included studies (risk of bias) using the JBI method for analytical cross-sectional study

**Author**	**Q1**	**Q2**	**Q3**	**Q4**	**Q5**	**Q6**	**Q7**	**Q8**	**Sum**
Ali et al. [19]	N	Y	Y	Y	Y	Y	Y	Y	7/8
Stewart et al. [20]	N	Y	Y	Y	Y	Y	Y	Y	7/8
Lin et al. [21]	U	Y	Y	Y	U	U	Y	Y	5/8
González-Agüero et al. [22]	U	Y	Y	Y	Y	Y	Y	Y	7/8
Jankowicz-Szymańska et al. [23]	N	Y	Y	Y	Y	Y	Y	Y	7/8
Izquierdo-Gomez et al. [24]	Y	Y	Y	Y	Y	Y	Y	Y	8/8
Pau et al. [25]	Y	Y	Y	Y	Y	Y	Y	Y	8/8
Real de Asua et al. [26]	Y	Y	Y	Y	Y	Y	Y	Y	8/8
Real de Asua et al. [27]	Y	Y	Y	Y	Y	Y	Y	Y	8/8
Romano et al. [28]	U	Y	Y	Y	Y	Y	Y	Y	7/8
Mansour et al. [29]	Y	Y	Y	Y	Y	Y	Y	Y	8/8
Calvo-Lobo et al. [30]	Y	Y	Y	Y	Y	Y	Y	Y	8/8
Pitchford et al. [31]	U	Y	Y	Y	Y	Y	Y	Y	7/8
Wolan-Nieroda et al. [32]	N	Y	Y	Y	Y	Y	Y	Y	7/8
Bibrowicz et al. [14]	N	Y	Y	Y	Y	U	U	Y	5/8
Magge et al. [33]	Y	Y	Y	Y	Y	Y	Y	Y	8/8
Suarez-Villadat et al. [33]	Y	Y	Y	Y	Y	Y	Y	Y	8/8
Sung et al. [34]	Y	Y	Y	Y	Y	Y	Y	Y	8/8
Herrera-Quintana et al. [35]	Y	Y	Y	Y	Y	Y	Y	Y	8/8
Ungurean et al. [36]	N	Y	Y	Y	Y	Y	Y	Y	7/8
Querido et al. [37]	Y	Y	Y	Y	Y	Y	Y	Y	8/8
Ungurean et al. [38]	N	Y	Y	Y	Y	Y	Y	Y	7/8

Q1- Were the criteria for inclusion in the sample clearly defined?; Q2- Were the study subjects and the setting described in detail?;Q3- Was the exposure measured in a valid and reliable way?; Q4- Were objective, standard criteria used for measurement of the condition?;Q5- Were confounding factors identified?;Q6- Were strategies to deal with confounding factors stated?;Q7- Were the outcomes measured in a valid and reliable way?;Q8- Was appropriate statistical analysis used?; *Y *yes, *N *no, *U *unsure, *NA *not applicable

## Results

### Study selection and characteristics

**Fig. 1:**
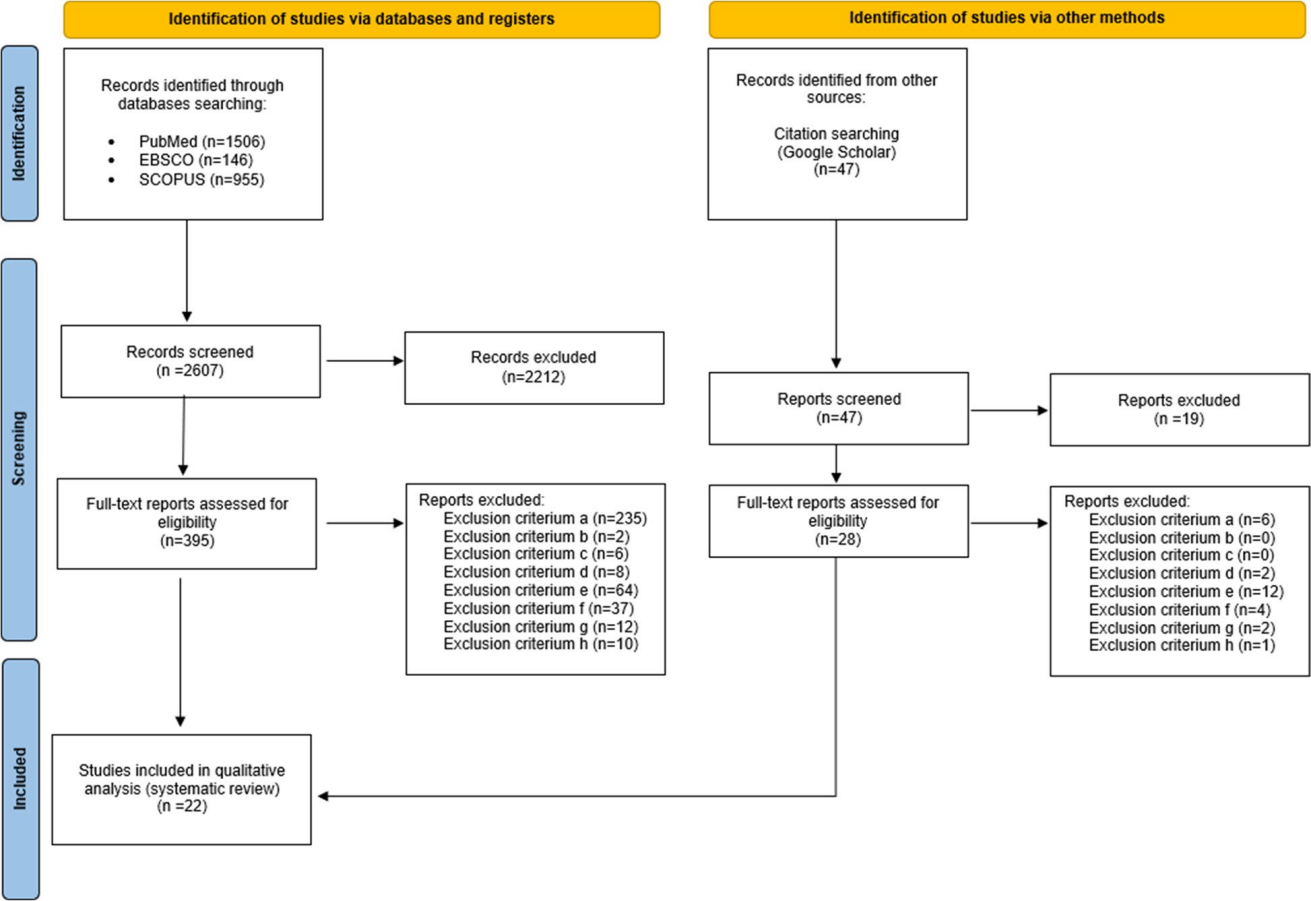
PRISMA flow diagram detailing the study inclusion process [17]

Figure 1 presents the flow of the systematic review. Three hundred ninety-five full-text articles were assessed to determine eligibility, while 22 studies met the inclusion criteria and were subjected to detailed analysis and assessment of their methodological quality (see Table 1).

Twenty-two reports that were assessed for their methodological quality were considered to score 8/8 points of eligibility to be included in the systematic review. Eleven publications were considered to score 8/8 points of eligibility, 9 scored 7/8 points of eligibility and 2 were assessed to score 5/8 points of eligibility. The initial agreement of the two independent investigators (EG, AZ) was 90%. All discrepancies among the investigators were resolved by expert evaluation by an independent co-author (DC). Finally, 22 full-text articles were included in the systematic review (see Tables 2, 3).

**Table 2:** The summary of the studies from 2006-2023 evaluating the characteristics of the body build and posture in individuals with DS

**Characteristics of the body build**						
**Author**	**Participants characteristics**			**Research** **Issue**	**Research** **Tool**	**Main findings**
	**Study participants**	**Study group**	**Control group**			
González-Agüero et al. [22]	nP=63; nF=27; nM=36	(DS individuals) nP=31; nF=14; nM=17/age=15.2±2.9	(Healthy individuals) nP=32; nF=13; nM=19/age=14.7±2.3	Comparison of the FM and LM between children and adolescents with and without DS and evaluation of the presence of sexual dimorphism	Stadiometer m (SECA 225, SECA, Hamburg, Germany) Weight (SECA 861, SECA, Hamburg, Germany) Anthropometric tape (Rosscraft, Canada) DXA BODPOD1 Body Composition System (Life Measurement Instruments, Concord, CA)	SG vs. CG WC,BMI, %BF - no significant differences were found between both groups (*p*>0.05). %BF, LM – females had higher rates than males from the respective group (*p*<=0.05) FM,LM - Higher values were observed in DS the trunk in females, while lower values were found in the lower limbs compared to females from CG (all *p*≤0.05). DS males had higher FM in the whole body and upper limbs, while lower values were observed according to LM in the whole body and lower limbs compared to males from CG (all *p*≤0.05).
Jankowicz-Szymańska et al. [23]	nP=80; nF=26; nM=54/age=18.68±1.73)	(DS individuals with mild disability – SG1) nP=40; nF=13; nM=27 (DS individuals with moderate disability – SG2) nP=40; nF=13; nM=27	N/A	Determination of the incidence of excessive body mass in DS adolescents	Tanita Anthropometer CQ Elektronik podoscope	Moderate DS female adolescents and mild DS male adolescents were found to have greater BM compared to the opposite intragender level of DS (9kg – females, 4kg – males). Both BMI and BM % were related to the level of DS disability in females (greater values at a moderate level)(*p*<0.03), which was not reported in males. 53.8% - DS females with overweight/obesity 15% - DS males with overweight/obesity The incidence of flat feet was found to be affected to a greater extent by the participants’ nutritional status than their degree of disability.
Izquierdo-Gomez et al. [24]	nP=111; nF=50; nM=61	(DS adolescents) nP=22; nF=5; nM=12/age=12-18	(Healthy adolescents) nP=94; nF=45; nM=49/age=12-16	Comparison of the level of fatness in adolescents with and without DS	ALPHA protocol for (anthropometry measurements)	SG vs CG 22.35±4.03 vs. 21.61±3.77 – BMI (*p*>0.02) 72.70±8.69 vs. 72.05±9.23 – WC (*p*>0.02) 10.56±3.79 vs. 16.20±9.77 – triceps skinfold 11.17±4.07 vs. 14.25±7.39 – subscapular skinfold 26.79±7.80 vs. 36.64±16.63 – BF % 48.80±9.87 vs. 58.34±13.08 – BM 147.65±7.76 vs. 163.85±8.07 – BH (*p*<0.001)
Real de Asua et al. [26]	nP=102; nF=49 nM=51/age=39.2±12	(DS adults) nP=51; nF=20; nF=31/age=36±11	(Healthy adults) nP=51; nF=31; nM=20/age=42.5±12.5	Determination of the prevalence of obesity in adults with DS and comparison to adults without DS	Stadiometer Calibrated balance scale	SG vs. CG: 25.5% vs. 61% - normal BM (*p*<0.001) 37% vs. 25.5% - overweight 37% vs. 14% - obesity 35% vs. 33% - abdominal obesity 86% vs. 68% - pathologic WHR (*p*<0.05)
Real de Asua et al. [27]	nP=98; nF=41; nM=48	(DS adults) nP=49; nF=20;nM=29/age=36±11	(Healthy adults) nP=49; nF=30; nM=19/age=42±13	Description of the anthropometric differences in weight-related disorders between adults with and without DS	Stadiometer Hand-held device (OMRON BF-306, OMRON Healthcare, Vernon Hills, IL, USA)	Higher prevalence of overweight, obesity, and WHR was found in the DS group compared to CG. SG vs CG: 28.8±4.7 vs. 24.9±3.8 – BMI (*p*<0.001) 26±10 vs.29±8 – total body fat 26% vs. 62% - normal BM (*p*<0.001) 37% vs. 24% - overweight 37% vs. 14% - obesity 18% vs. 18% - abdominal obesity 93.5±12.1 vs. 90.0±12.2 – WC 0.62±0.09 vs. 0.54±0.08 – WHR (*p*<0.001)
Pitchford et al. [31]	nP=39; nF=18; nM=21	(DS adolescents) nP=22; nF=8; nM=14/age=14.96±1.92	(Healthy adolescents) nP=17; nF=10; nM=7/age=15.08±2.12	Comparison of the group differences between adolescents with and without DS	Whole-body DXA scan (GE Lunar Prodigy Advance [DPX-IQ 240] densitometer; Lunar Radiation Corp, Madison, WI)	Adolescents with DS were found to have significantly higher BMI (*p* = .001, *d* = 1.06), BMI percentile based on growth charts (*p* < .001, *d* = 1.31), and %BF (*p*<0.05) compared to CG. Significant differences were also found between groups in total %BF (*p* = .011), fat in regional segments at the arms, legs, and trunk (*p* < .020), and for body fat ratios of trunk-to-total, legs-to-total, and arms and legs-to-trunk (*p* < .001, *d* >1.15).
Magge et al. [33]	nP=253; nF=145 ;nM=108	(DS adolescents) nP=150; nF=84; nM=66/age=14.4	(Healthy adolescents) nP=103; nF=61; nM=42/age=14.8	Understanding of the relationship between BMI and more direct measure of adiposity in adolescents with and without DS compared by age, gender, race, ethnicity, BMI percentile	Digital electronic scale (Scaletronix) Wall-mounted stadiometer (Holtain) DXA	64% of DS adolescents - BMI percentile ≥85 DS adolescents had lower values of VF, FM, and LM based on BMI score, and greater VF at higher FM compared to CG.
Suarez-Villadat et al. [33]	nP=263 females and males	(DS adolescents) nP=100; nF=36; nM=64/age=15.7±2.4	(Healthy adolescents) nP=163 females and males/age=13.8±1.4	Examination of the changes in several indicators of the body composition (BMI, WC, WHR, TS, BF(%), SS) in adolescents with and without DS	Electronic scale (model SECA 701, Hamburg, Germany) Telescopic height-measuring instrument (model SECA 220) Nonelastic tape(SECA 200; SECA) Holtain caliper	Baseline assessment SG vs. CG 36% vs. 9% - overweight/obesity 23.6±4.1 vs. 20.9±3.3 – BMI 73.2±9.7 vs. 68.5±7.1 – WC 34.2±12.8±19.7±9.4 - %BF 2-year follow-up assessment; the following changes were noted between SG vs. CG: 24.8±4.2 vs. 21.8±3.3 - decrease in BMI (*p*<0.05) 30.6±11.6 - decrease in %BF (only in DS adolescents)(*p*<0.05)
Herrera-Quintana et al. [35]	nP=23; nF=10; nM=13/29.4±5.95	(DS individuals) nP=23; nF=10; nM=13/29.4±5.95	N/A	Analysis of the anthropometrical and body composition profiles of participants with DS	Stadiometer (Seca, model 213, range 85–200 cm; precision: 1 mm; Hamburg, Germany) Tanita MC-980 Body Composition Analyzer MA Multifrequency Segmental, (Barcelona, Spain) Height rod	Females with DS were characterized by higher LM (*p*<0.001), FM (*p*<0.001), bone mass (*p*<0.01), and WHR (*p*<0.01) compared to males with DS. Both LM and bone mass were found to decrease and BMI, FM, and WHR were found to increase with age in both genders (*p*<0.05).
Querido et al. [37]	nP=37 females and males	(DS swimmers) nP=18; nF=4; nM=14/age=22.2±5.4	(DS untrained subjects) nP=19; nF=10; nM=9/age=26.6±8.2	Assessment of the body composition of DS competitive swimmers and untrained DS subjects	Harpender skinfold caliper Electronic weighing scale)	SG1 vs. SG2 44.4% vs.52.6% - incidence of overweight/obese 51.5±16.5 vs. 81.9±27.9 – skinfold sum 25.4±3.8 vs. 30.7±10.2 – BMI 21.2±6.0 vs. 29.8±7.1 - % BF 22.1±4.2 vs. 20.05±3.7 – lean BM 9.3±3.5 vs. 13.6±6.1 – FM index
**Characteristics of the body posture – feet**						
Pau et al. [25]	nP=118 females and males	(Male, obese DS individuals – SG1) nP=34/age=12.3±2.7 (Female, obese DS individuals – SG2) nP=25/age=12.7±2.7	(Male, DS individuals with normal body mass– CG1) nP=34/age=12.8±2.7 (Female, DS individuals with normal body mass– CG2) nP=25/age=12.3±3.1	Characterization of the effect of obesity on foot-ground contact in young DS individuals during quiet upright stance	Pressure-sensitive mat (Tekscan Inc, South Boston, MA)	In both male and female DS children, flatfoot was found to be the predominant arch type that simultaneously was not related to obesity. Obesity was found to impact on the foot-ground interaction, which was higher in female DS children (larger contact areas and higher plantar pressure in the fore/midfoot). The modifications induced by obesity in foot-ground contact pressure and area may aggravate the existing foot disturbances in DS children.
Mansour et al. [29]	nP=108; nF=45; nM=63	(DS individuals) nP=55; nF=19;nM=36/mean age=14	(Healthy individuals) nP=50; nF=26;nM=27/mean age=11	Investigation of the prevalence of known foot deformities in patients with DS and of other previously non-described foot anomalies in DS population	Podoscope (Podometer, Pedi-health Ltd)	Prevalence of foot deformities between DS and CG groups 36.4% vs. 6.6% - incidence of hallux valgus (*p*<0.001) 73.6% vs. 2.8% - increased space between the 1^st^ and 2^nd^ toes (*p*<0.001) 17.3% vs. 0% - incidence of both hallux valgus and increased space between the 1^st^ and 2^nd^ toes (*p*<0.001) 12.7% vs. 0% - incidence of syndactyly (*p*<0.001) 15.5% vs. 4.7% - incidence of clinodactyly (*p*<0.001) 39.1% vs. 15.1% - pes planus grades II (*p*<0.001) 30% vs. 2.8% - pes planus grades III (*p*<0.001)
Calvo-Lobo et al. [30]	nP=105/age=35.71±12.93	(DS group) nP=50/age=25.58±8.16	(Healthy controls) nP=55/age=44.92±8.95	Evaluation of the foot conditions of persons with DS and determination of wearing the suitable footwear	Brannock Device-type measuring instrument	92% of DS individuals had foot problems, among which the following were the most common: flat foot (92%), hyperkeratotic lesions, muscle and ligament laxity (78%), hypotonia (73%), hypermobility of the first ray (52%), nail lesions (52%), metatarsus primus adductus (48%). The prevalence of flatfoot in the CG group was found to correlate with the presence of joint laxity, which was not reported in the DS group. 76% of DS individuals wore inadequate footwear.
**Characteristics of the body posture – spinal curvatures**						
Ali et al. [19]	nP=44; nF=15; nM=29/age=26.64±8.46	DS individuals were selected based on age criterion: (SG1) nP=11/age=15-19 (SG2) nP=20/age=20-29 (SG3) nP=9/age=30-39 (SG4) nP=4/age=40-45	N/A	Assessment of the cervical spine abnormalities that are related to DS	Radiograph	36% - cervical spondylosis 18% - atlantoaxial instability 12% - congenital anomalies of C1-C2. Degenerative changes in the cervical spine were found to increase with age, mostly in the cervical levels, and to occur earlier than in the healthy population.
Romano et al. [28]	nP=40 females and males	(Down syndrome individuals) SG=30; nF=12; nM=18/mean age=16	(Healthy volunteers) nP=10 females and males	Assessment of the kind of information provided by MRI examination in spinal flexed and extended position in DS patients with cranio-cervical instability	1,5 Tesla Magnet (PHILIPS®, Intera, 1,5 T, The Netherlands), and a Neck-Array Coil.	DS patients had smaller values of SAC (*p*<0.02) and greater values of ligament thickness (*p*<0.001) compared to health CG. A significant reduction of ASAS in flexed position was evident in DS subjects compared to CG in neutral (*p*<0.001) and flexed (*p*<0.002) positions.

*ASAS* Anterior subarachnoid space, *BF* Body fat, *BM* Body mass, *BMI* Body mass index, *CG* Control group, *DS* Down syndrome, *FM* Fat mass, *LM* Lean mass, *MRI* Magnetic resonance imaging, *N/A* Not applicable, *nF* Number of females, *nM* Number of males, *nP* Number of participanrs, *SAC* Space available for the cord, *SG* Study group, *SS* Scapula skinfold, *TS* Triceps skinfold, *VF* Visceral fat, *WC* Waist circumference, *WHR* Waist to hip ratio

**Table 3:** The summary of the studies from 2009-2023 evaluating the characteristics of the body build and posture in individuals with ID excluding DS population

**Characteristics of the body build**						
**Author**	**Participants characteristics**			**Research** **Issue**	**Research** **Tool**	**Main findings**
	**Study participants**	**Study group**	**Control group**			
Steward et al. [20]	nP=206; nF=56/age=14.1±3.8; nM=150/age=13.1±3.3	nP=206; nF=56/age=14.1±3.8; nM=150/age=13.1±3.3	N/A	Estimation of the obesity prevalence in ID children and comparison with the population prevalence	Leicester Height Meter (Child Growth Foundation, London, UK) SECA Alpha Scale (Child Growth Foundation, London, UK)	Prevalence of obesity was found to be significantly higher in ID children than in the general population (*p*< 0.01).
Sung et al. [34]	nP=428 females and males	(Typical development children – SG1) nP=355; nF=172; nM=183/age=11-12 years old (Children with ID – SG2) nP=73; nF=23; nM=50/age=11-12 years old	N/A	Comparison of the growth and body composition between typical development children and children with ID	Inbody 770 and Inbody S10 (InBody Co., Ltd, Seoul, Korea)	Children with ID were found to have significantly lower fat mass (*p*<0.05) compared to SG1. The rates of the variables of body build and composition were smaller in children with ID compared to SG1.
Ungurean et al. [36]	nP =101 males and females	(Males without ID – SG1) nP=23/age=17.7±0.9 (Females without ID – SG2) nP=26/age=17.2±0.9 (Males with moderate ID – SG3) nP=34/age=16.4±0.9 (Females with moderate ID – SG4) nP=12/age=16.2±0.1 (Males with severe ID) nP=6/age=16.8±0.9	N/A	Identification of the parameters of body composition that impact on the values of BMI in children with and without ID	Telemeter with a Bosch GLM 80 laser TANITA MC 580 S	Both gender groups of ID children were characterized by similar prevalence of excess BM and obesity. ID boys were characterized by more frequent prevalence of obesity (BMI>24) compared to overweight (BMI rating between 21.5 – 24).
Ungurean et al. [38]	nP=212 females and males/age=17.7±0.2	(Males without ID – SG1) nP=44/age=17.7±0.9 (Females without ID – SG2) nP=55/age=17.2±0.7 (Males with moderate ID – SG3) nP=57/age=17.05±0.7 (Females with moderate ID – SG4) nP=22/age=16.6±0.8 (Males with severe ID) nP=23/age=17.4±0.8 (Females with severe ID) nP=11/age=17.1±0.8	N/A	Assessment of the body composition between adolescents with and without ID	Telemeter with a Bosch GLM 80 laser TANITA MC 580 S	BM, BMI, BF (%,kg), MM (%,kg), BMR, SMM, BH were found to be likely to be influenced by both gender and level of disability. %BF was found not to be influenced by the level of ID.
**Characteristics of the body posture – feet**						
Wolan-Nieroda et al. [32]	nP=90/ mean age=11.49±2.3	(Individuals with ID) nP=45	(Healthy individuals) nP=45	Assessment of the parameters of foot shape in children and adolescents with ID Examination of the relationship between the degree of disability and the relevant foot parameters	CQ-ST apparatus manufactured by Electronic System, Poland	Children with mild ID were characterized by the greater length of the right (*p*=0.006) and left (*p*=0.004) foot and Wejsflog's rate for the right (*p*<0.001) and left (*p*<0.001) foot. Higher values of the gamma angle of the right (*p*=0.028) and left (*p*=0.006) foot were noted in children with moderate ID. Healthy individuals were more often characterized by healthy foot structure (Cook’s angle).
**Characteristics of the body posture – spinal curvatures**						
Lin et al. [21]	nP=822; nF=323; nM=499/age=15.69±0.75	nP=822; nF=323; nM=499/age=15.69±0.75	N/A	Analysis of the conditions of spinal and limb abnormalities in adolescents with ID and examination of their predictors	X-ray	14.5% - spinal abnormalities 8.6% - limb abnormalities Spinal abnormalities were correlated with BMI (*p*<0.001) and limb abnormalities (*p*<0.001). Underweight (based on BMI) increased the risk of spinal abnormalities (*p*<0.05).
Bibrowicz et al. [14]	nP=30	(Individuals with DS) nP=20/age=22-34 years old	(Healthy individuals) nP=10/age=17-34	Evaluation of the posture quality in volleyball players with and without ID	Photographic Postural Assessment System manufactured by the OPIW company (Opolskie Przedsiębiorstwo Innowacyjno-Wdrożeniowe)	SG vs CG 73% vs. 40% – sway back posture 20% vs. 30% - increased lumbar lordosis 40% vs. 100% - harmonious posture 20% vs. 0% - faulty posture 40% vs. 0% - bad posture

*BF* Body fat, *BH* Body height, *BM* Body mass, *BMI* Body mass index, *BMR* Basal metabolic rate, *CG* Control group, *ID* Intellectual disability, *MM* Muscle mass, *N/A* Not applicable, *nF* Number of females, *nM* Number of males, *nP* Number of participanrs, *SG* Study group, *SMM* Skeletal muscle mass

## Discussion

A careful examination of the current scientific studies on the body build and posture in the population of ID individuals, including DS has yielded partially inconsistent findings. However, this qualitative analysis found gender, age, and level of ID to be the most frequent factors that impact the intrinsic differences in body build and posture and the occurrence of their disturbances in DS individuals compared to other people with ID (see Table 2).

The majority of the analyzed studies have found several statistically significant relationships between sexual dimorphism and the abnormalities of the qualities and indicators of body build and postural disorders in DS individuals [22, 23, 25]. This was also confirmed by the study of Herrera-Quintana et al. [35], but a 2-year follow-up examination indicated a similar tendency for an increase in indicators of body build such as BMI and WHR and decrease in lean mass and bone mass in both genders [35]. On the contrary, the study conducted by Querido et al. [37] reported a similar prevalence of changes in the body build and posture in both males and females with DS. The inconsistencies in the results of the abovementioned research can be explained mainly by differences in the characteristics of participants presented in the qualitative analysis of this systematic review i.e., (1) internal compensatory mechanisms induced by the internal variables such as (a) level of ID, (b) gender, (c) age, (d) intrinsic characteristics of the morpho-functional development, (e) body mass, (f) ligament laxity and joints mobility, and (2) external mechanisms induced by the following external variables i.e., (a) type of sport, (b) training load (years of training/ number of training sessions per week), and (c) footwear.

Numerous authors have suggested that the proximodistal development that is related to DS differs by gender (see Table 2). Some studies have indicated better parameters of body build (higher lean mass and bone mass) in DS females [22, 23, 35], while Pau et al. [25] have found more disturbances in body build and posture (feet) in DS females compared to DS males. Nevertheless, the majority of the analyzed studies [24, 26, 31, 33] reported body qualities (BF, BH, BM, WC, visceral fat) and indicators (BMI, WHR, visceral fat rate) as variables that are related to the intrinsic characteristics of the morpho-functional development that is specific to DS individuals. However, Real de Asua et al. [27] found a similar prevalence of abdominal obesity in both DS individuals and healthy controls, while lower values of total BF were reported in the DS group, and González-Agüero et al. [22] reported on diversity in the location of the FM between males and females with DS. The uncertainty of the cited studies indicates the complexity of the body build variability that seems to depend both on gender and individual morpho-functional development. Furthermore, there is a need to indicate the importance of internal compensatory mechanisms that affect fat mass distribution in DS individuals, which in turn was found to impact intrinsic body composition. This is consistent with our previous research on the lipid profile of ID patients [39].

This qualitative analysis suggested that body build and posture are impacted by both level of ID and age of DS individuals [19, 23, 33, 35]. Interestingly, the study by Jankowicz-Szymańska et al. [23] reported a significant relationship between body build qualities (BM) and indicators (BMI) and the level of ID. However, the abovementioned variables were found only in DS females. On the contrary, age was reported to impact body build and posture similarly in both genders [19, 33, 35], mainly by decreasing the density of the musculoskeletal structures [35]. Moreover, the incidence of the anatomical differences in the cervical spine between DS individuals and those from the ID population that occurred with age seem to significantly contribute to the degenerative changes in the cervical level of the spine, especially as cervical spondylosis [19]. This might be a result of the disadvantageous impact of the internal compensatory and adaptive changes in the upper segments of the spinal curvatures (internal compensatory mechanism). This thesis was also confirmed by Romano et al., [28], who reported a decrease in SAC and ASAS in DS adolescents that were related to the ligament thickness.

Calvo-Lobo et al. [30] also indicated a relationship between joint laxity and foot deformities but the correlation was confirmed only in healthy adults. However, hypermobility of the first nail was reported as a factor affecting the incidence of flatfoot in DS adults [30]. Similar findings were reported by Pau et al. [25] who found a dominant tendency for flatfoot in male and female DS adolescents, simultaneously indicatingno relationship between foot deformities and overweight. These findings are also consistent with the study by Mansour et al. [29], who reported a high prevalence of foot deformities in DS adolescents, which occurred especially as hallux valgus and increased space between the 1^st^ and 2^nd^ toes, suggesting the relationship with morpho-functional development of DS patients.

Even though internal compensatory mechanisms induced by the abovementioned factors seem to be the crucial determinants of the special characteristics and disturbances of the body build and posture in DS individuals, the impact of external variables should also be indicated. As reported in the study by Calvo-Lobo et al. [30] inadequate footwear, which was found in the majority of DS adults, could be related to the deepening of the foot deformities. Nevertheless, external variables were also reported to contribute to the body build of DS individuals. The study by Querido et al. [37] showed that swimming training had a beneficial effect on the somatic parameters of body build by contributing to an increase in LM and a decrease in BF (%) and BMI.

Based on a detailed review of the current scientific reports (Table 3) it is difficult to confirm the direct effect of the intrinsic variables that impact the body build and posture in the ID population, excluding people with DS. For instance, some studies [32, 36, 38] have reported a relationship between the level of ID and body build and posture. On the contrary, other reports have not identified the crucial factor that may be related to the incidence of disturbances in the body build and posture in the ID population [14, 20, 34]. The inconsistent results of the presented reports can be explained, similar to those on DS individuals, by differences in the characteristics of participants, including intrinsic variables that might induce internal compensatory mechanisms i.e., (a) level of ID, (b) gender, (c) lower limbs dysfunctions, (d) body mass, and (e) BMI.

The study by Ungueran et al. [36] reported a significant relationship between the level of ID and excessive BM and the prevalence of overweight in male and female adolescents with ID. However, no relationship was found for gender. Similar findings were obtained by Stewart et al. [20], who indicated a high prevalence of overweight and obesity in ID adolescents. Nevertheless in another study by Ungueran et al. [38], both of the abovementioned relationships were noted, which may suggest that the level of ID may be the predominant variable related to the body build in the ID population. Similar findings were concluded by Sung et al. [34], who suggested ID as a factor in delayed and disturbed body build. However, Lin et al. [21] indicated both underweight (based on BMI) and lower limb dysfunctions as the factors affecting the incidence of spinal curvature disturbances in the ID population. This might be attributable to the internal compensatory mechanism leading to different body build and posture in people with DS compared to the population of people with ID.

### Limitations and strengths

While this qualitative analysis contributes to the current body of literature, there are some limitations that need to be addressed. The main limitation of the current study is the small number of studies that have investigated the ID population, which did not allow for general interference. Moreover, the evaluation of the body build and composition in DS and ID populations was performed using different methodologies, which makes generalization impossible. Nevertheless, it should be acknowledged that the research with participation of intellectually disabled participants is highly difficult and the number of DS and ID individuals that can be included in the studies is limited.

The main strength of the present study is the systematic review of the latest reports from the last two decades that have examined the body build and posture in DS and ID populations. Furthermore, the majority of the included studies were evaluated to be perfectly eligible for this analysis. The authors believe that the novelty of the presented research problem and undertaking the hitherto unexplored aspects in scientific research will enable a better understanding of the limitation of the kinesthetic abilities of people with ID and DS. It might also help improve and optimize the education and rehabilitation programs in the populations of people with DS and other ID using direct stimulation based on physical activity focused on their biomechanical and morpho-functional strengths.

## Conclusions

1. The presented systematic review found that compared to other ID, the intrinsic differences in the body build and posture in DS individuals were induced mainly by gender, age, and the level of ID.
2. The conducted qualitative analysis indicates a tendency for diversity between DS individuals and other ID populations in body build and posture that are determined by the presence of the third copy of chromosome 21 in the former group.
3. Internal compensatory processes may be induced mainly by abnormalities in the structure of the cervical vertebrae and feet.
4. IQ should not be used as the only variable that identifies the population of people with ID.

## Data Availability

The data collected and analyzed during the current study are available from the corresponding author on reasonable request.

## Abbreviations

DS: Down syndrome
ID: Intellectual disability
PRISMA: Preferred reporting items for systematic reviews and meta-analyses
JBI: Joanna Briggs Institute
ASAS: Anterior subarachnoid space
BF: Body fat
BM: Body mass
BMI: Body mass index
CG: Control group
FM: Fat mass
LM: Lean mass
MRI: Magnetic resonance imaging
N/A: Not applicable
nF: Number of females
nM: Number of males
nP: Number of participanrs
SAC: Space available for the cord
SG: Study group
SS: Scapula skinfold
TS: Triceps skinfold
VF: Visceral fat
WC: Waist circumference
WHR: Waist to hip ratio
BH: Body height
BMR: Basal metabolic rate
MM: Muscle mass
SMM: Skeletal muscle mass
